# Unusual and Unconsidered Mechanisms of Bacterial Resilience and Resistance to Quinolones

**DOI:** 10.3390/life14030383

**Published:** 2024-03-14

**Authors:** Joaquim Ruiz

**Affiliations:** Grupo de Investigación en Dinámicas y Epidemiología de la Resistencia a Antimicrobianos—“One Health”, Universidad Científica del Sur, Lima 15067, Peru; joruiz.trabajo@gmail.com or jruizb@cientifica.edu.pe

**Keywords:** amoeba, quinolone targets, biofilm, stringent response, toxin/antitoxin systems, quinolones inactivation, RNA polymerase mutation

## Abstract

Quinolone resistance has been largely related to the presence of specific point mutations in chromosomal targets, with an accessory role of impaired uptake and enhanced pump-out. Meanwhile the relevance of transferable mechanisms of resistance able to protect the target of pump-out or inactivate quinolones has been increasingly reported since 1998. Nevertheless, bacteria have other strategies and mechanisms allowing them to survive and even proliferate in the presence of quinolones, which might be qualified as resistance or resilience mechanisms. These include decreasing levels of quinolone target production, transient amoeba protection, benthonic lifestyle, nutrient-independent slow growth, activation of stringent response, inactivation or degradation of quinolones as well as apparently unrelated or forgotten chromosomal mutations. These mechanisms have been largely overlooked, either because of the use of classical approaches to antibiotic resistance determination or due to the low increase in final minimum inhibitory concentration levels. This article is devoted to a review of a series of these mechanisms.

## 1. Introduction

**Figure 1:**
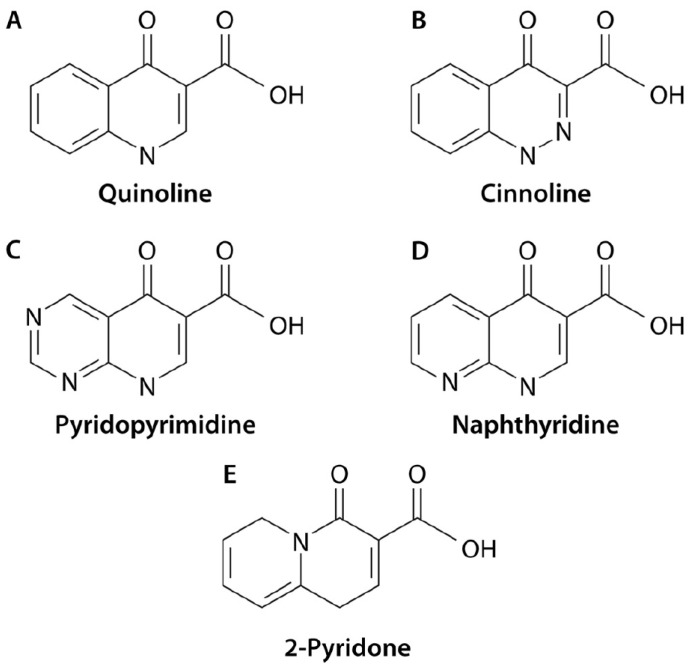
Subclasses of quinolones. At present, 4 subclasses of quinolones (**A**) quinolines; (**B**) cinnolines; (**C**) Pyridopyrimidine; (**D**) Naphthyridine have been described; atom numeration is described following the quinolines (**A**) structure, with position 1 risen in the nitrogen atom. The figure also illustrates the structure of 2-Pyridons (**E**), because they are structurally related, but at present, none have been introduced in human or veterinary practice. Reproduced from reference [1], with permission from the American Society of Microbiology.

Quinolones are fully synthetic molecules which may be classified within four different main subgroups as regards the position of nitrogen atoms in the molecule [1] (Figure 1).

These antimicrobial agents are able to inhibit DNA synthesis, transcription and cell division through their interaction with Type II Topoisomerases (i.e., DNA Gyrase and Topoisomerase IV) [2,3]. Thus, quinolones bind to the complex DNA-Topoisomerase blocking the replication forks [4], with these findings leading to bacterial kill through different events [3].

Despite the presence of previous reports [5,6,7], the official history of quinolones as antimicrobial agents is considered to have started in 1962 with the description of nalidixic acid [8]. Although several studies reported the use of nalidixic acid for the treatment of infections such as diarrhea or dysentery [9,10,11], nalidixic acid as well as ancient quinolone therapeutic uses were almost limited to fighting urinary tract infections [12]. In the following years, the family of quinolones grew exponentially, and with the incorporation of a fluorine atom in position 6, their uses expanded from human urinary infections to a great variety of uses, including those in human therapeutics, such as dermic, gastrointestinal, ocular, osteoarticular, respiratory, skin and soft-tissue and systemic infections, among others [13,14,15,16,17,18,19,20,21]. In the 2000s, a series of quinolones lacking a fluorine atom at position 6 were synthesized, with several of them, such as ozenoxacin and nemonoxacin introduced in clinical practice [22,23]. Quinolones have also been used in the treatment of sick animals or for prophylactic purposes [24,25,26,27], in addition to use as livestock growth promoters, despite the potential side effects on cartilage [28]. Furthermore, apart from therapeutic uses related to bacterial pathogens, the use of quinolones against fungi, viruses and parasites has also been explored [29,30,31,32].

These expansive uses have led to an increasing isolation of quinolone-resistant microorganisms, resulting in a worldwide epidemic of quinolone resistance from the mid-1990s onward [1]. Subsequently, quinolone resistance levels, selection, mechanisms, and dispersion pathways have been largely and increasingly studied since the 1960s [21,33,34,35,36,37,38,39,40,41,42,43,44,45,46,47].

In the late 1970s, the implication of GyrA in the development of quinolone resistance was observed [48]. The relevant role of punctual mutations in quinolone targets encoding genes (*gyrA* and *gyrB*, encoding DNA-Gyrase, and *parC* and *parE*, encoding Topoisomerase IV), able to avoid or limit the ability of quinolones to interact with their targets, was clearly established in the 1980s and 1990s and was subsequently considered a universal pathway of the development of quinolone resistance in both Gram-negative or Gram-positive bacteria, including particular microorganisms, such as *Mycobacterium* spp. [21,34,46,49,50,51,52,53,54,55,56]. Furthermore, it was described that several microorganisms presenting wild-type-specific amino acids in critical positions of GyrA and/or ParC showed natural resistance to quinolones [57,58]. In parallel, the role of efflux pumps and impaired permeability was also demonstrated, being considered as responsible for the basal minimal inhibitory concentration (MIC) levels of quinolones and an accessory mechanism of the development of quinolone resistance, when specific mutations lead to down-expression of porins or overexpression of efflux pumps [45,55,59,60,61,62]. The role of alterations in quinolone uptake has been considered as the most relevant route of quinolone resistance acquisition only in specific microorganisms, such as *Stenotrophomonas maltophilia* [63,64], while target mutations seem to play a residual role [64,65,66].

Despite the publication of previous unconfirmed or misinterpreted studies [1,37,67,68,69,70,71], the first undoubtable transferable mechanism of quinolone resistance (TMQR) was described in 1998 [39], with different TMQRs being reported in the following years and classified into three main generic mechanisms, quinolone target protection (*qnr*), quinolone inactivation/modification (*aac(6′)Ib-cr* and *crpP*) and quinolone pump-out (*qepA*, *oqxAB* and others), overall accounting for more than 12 different genes and hundreds of allelic variants [1,72,73,74,75,76]. Of note, the role of CrpP as a mechanism of quinolone resistance has recently been questioned, and it has been proposed to not consider it among TMQRs [77].

These well-established and largely studied mechanisms of quinolone resistance are referred to as “canonical mechanisms” in the present text. The vast majority of studies on quinolone resistance mechanisms, as well as specific thematic reviews, are focused on what was mentioned above [1,42,45,46,49,55,60], while the approaches on acquisition and dispersion of quinolone resistance mostly describe the selection of target mutations as well as the role of mobile (or mobilizable) genetic elements, such as plasmids, transposons, genomic islands, and integrons [1,44,76,78,79,80,81,82]. Nonetheless, in addition to these well-established mechanisms of resistance to quinolones, several less frequent mechanisms as well as non-classical routes of acquisition of quinolone resistance or quinolone tolerance have been reported in the literature, but they seem to have fallen by the wayside and are not considered (Table 1). The present review is focused on these less-studied mechanisms.

From early studies focused on the analysis of the mechanisms of resistance to quinolones, the presence of unexplained quinolone-resistant bacteria or microorganisms with discordances between quinolone resistance levels and reported mechanisms of quinolone resistance has been frequent. While the acquisition of new knowledge has allowed sequential identification of new mechanisms of quinolone resistance in these unexplained quinolone-resistant strains, which often become a frequent motif of further studies and are classified as new canonical mechanisms of quinolone resistance [1,55], a series of minority or mostly neglected quinolone resistance mechanisms are present in the literature. In addition, although they are not strictly resistance mechanisms, several pathways also drive to increased survival in the presence of quinolones.

In the next sections, a series of pathways (Table 1) leading to increased bacterial survival in the presence of quinolones are presented in alphabetical order; therefore, no conclusion about higher or lower relevance may be inferred for the order in which they are exposed within the text.

## 2. Altered Production of Quinolone Targets

While target overexpression has been considered a mechanism of resistance to different antibacterial agents because the quantity of the target surpasses the inhibitory capacity of antibiotics, with overexpression of β-lactam inhibitor-sensitive TEM-1 leading to intermediate-to-full resistance to β-lactam plus β-lactam inhibitor combinations being a classic example [83,84,85], quinolones show an opposite phenomenon: lower levels of quinolone targets lead to higher levels of resistance. This phenomenon was first described in an in vitro selected quinolone-resistant *Staphylococcus aureus* in 2003 [86]. Using premafloxacin, Ince et al. selected an in vitro mutant exhibiting a 4-fold increase in the MIC levels of premafloxacin (from 0.004–0.008 mg/L to 0.016–0.032 mg/L) and ciprofloxacin (from 0.125–0.25 mg/L to 1 mg/L), with no alterations in either the MICs of ethidium bromide (suggesting no overexpression of efflux pumps) or amino acid substitution in GyrA, GrlA (ParC), GyrB or GrlB (ParE), and with a single nucleotide change G→A in the *grlBA* operon promoter at position -13, just downstream of a presumptive Shine–Dalgarno sequence [86]. The role of this alteration in the development of quinolone resistance was demonstrated by introducing the mutation in a wild-type *S. aureus*, confirming the increase in ciprofloxacin and premafloxacin MIC levels [86]. When the authors determined the physiological effects of this alteration, they observed a 3-fold reduction in the overall expression levels of *grlA* and *grlB* [86]. Nevertheless, no apparent effect on bacterial growth was observed, suggesting the presence of compensatory mutations within the chromosome [86].

The presence of alterations in the *gyrA* promoter region impacting sensitivity and supercoiling regulation and altering final expression levels has also been described [87], but no data on the effect on final quinolone levels are available in the literature. Of note, quinolones relax DNA by inhibiting supercoiling [88], which, in turn, results in increased expression of *gyrA* [89].

In relation to the above, potential alterations in the level of expression of genes encoding quinolone targets have been proposed as one of the mechanisms involved in the increased levels of resistance of the so-called “Small Colony Variants” (SCVs) [90] (see Section 8: Unconsidered Chromosomal Mutations).

While to the best of my knowledge no description has been made, it could be hypothesized that mutations at the initial codon of genes encoding quinolone targets leading to modifications from ATG to, for instance, GTG or TTG, may also affect transcription efficiency, as these initial codons are less efficient [91], thereby resulting in lower protein levels and subsequent similar effects on the final quinolone susceptibility levels.

## 3. Amoeba Protection

Free-living amoebae are potential pathogenic environmental microorganisms which live in water or soil environments predating bacteria communities [92]. Nonetheless, two singularities have been observed in the amoeba/bacteria relationships. These are the presence of a series of endosymbiotic bacteria, which in several cases seem to be unable to live in an amoeba-independent form [92], as well as the ability of several predated bacteria (including relevant pathogens, such as *Legionella pneumophila* or *Pseudomonas aeruginosa*) to survive and proliferate inside amoebae according to transient adaptations [93,94]. Thus, the amoeba environment might act both as an additional barrier for quinolones (as for other antimicrobial agents) to access bacteria, because of the need of antibacterial agents to cross the amoeba cell membrane, as well as directly influencing the bacterial transcriptome. Furthermore, the ability of bacteria to exchange genetic material inside amoebae has been demonstrated [95].

In addition to the effects on quinolone resistance, the presence of microorganisms inside amoebae may have additional effects on human (and animal) health, acting as a reservoir of pathogens and becoming a neglected route for bacterial infections [96].

It is necessary to highlight that, in the present text, due to the scarcity of data on amoebae, a series of assumptions based on macrophages data have been formed.

### 3.1. Barrier Effect?

While quinolones may penetrate inside eukaryotic cells, the presence of quinolones within amoeba cytoplasm may be affected by the need to cross the border membrane and the presence of amoeba efflux pumps, with possible interspecific and/or intraspecific differences. While this finding seems intuitive, no article referring to the accumulation of quinolones inside amoebae has been found in the literature. This lack of data may be related to the good intracellular penetration of quinolones, which has been observed in macrophages, among other eukaryotic cell types [97,98], and with the observed anti-amoeba activity of fluoroquinolones [99]. Nonetheless, the presence of eukaryotic efflux pumps able to pump out quinolones has been highlighted [100]. Furthermore, the contribution of eukaryotic efflux pumps to intracellular bacteria survival in the presence of fluoroquinolones has been described [101]. In this regard, while no data on quinolone efflux in amoebae have been found in the literature, the abovementioned quinolone extrusion mediated by efflux pumps in eukaryotic cells suggests that the presence of similar mechanisms in amoebae is highly probable [102]. Thus, in the absence of specific data, a barrier effect, which might be amoeba-species- or amoeba-strain-dependent and affect quinolones in a selective manner, cannot be discarded.

A special situation is amoeba encystment. Encystment is a defensive strategy in which the amoeba remains inactive, in a resting form, allowing amoeba survival in special adverse situations, such as the presence of toxins, including several produced by microorganisms such as *P. aeruginosa*, starvation, dehydration or osmotic stress, among others [103,104]. Amoeba cysts possess an external layer, which varies from species to species and has been considered as a reason to explain the higher resistance to common treatments exhibited by amoeba cysts in comparison with trophozoites [103]. Of note, it has been observed that different amoeba-resident bacteria, such as *Mycobacterium avium*, may surpass the cyst period, thereby also remaining under the protective umbrella of the cyst layer during the cyst period [105]. While ciprofloxacin has shown activity against amoeba cysts [99], it is likely that the cyst layer contributes to hindering its access to the amoeba cytoplasm, especially in environments with low quinolone concentrations.

### 3.2. Effects on Bacterial Transcriptome

In 1995, when analyzing the effect of *L. pneumophila* grown inside amoebae and macrophages on the final bacterial MIC and its survival levels in the presence of antibiotics, Barker et al. observed that in *L. pneumophila* released from amoeba, the MIC of ciprofloxacin did not increase with respect to in vitro cultured *L. pneumophila* (MIC = 0.06 µg/mL). Nevertheless, when exposed to 1 µg/mL of ciprofloxacin, survival rates increased 1000-fold [106]. Of note, after 48 h of in vitro growth, the survival ability of amoeba-grown *L. pneumophila* was lost [106]. No analysis of the mechanisms of ciprofloxacin survival acquired was performed, but the authors observed deep morphological changes including motility affectation [106]. Although no analysis of quinolone targets was performed, all these findings strongly suggest a transient adaptative response and not a true quinolone-resistant mutant selection. Similarly, studies analyzing the intracellular activity of ancient quinolones against *Francisella noatunensis* subsp. *noatunensis* or *Burkholderia mallei* have shown the inability of flumequine to inhibit *F. noatunensis* replication, despite high flumequine intake within macrophages [98], and the inability of flumequine to completely clear *B. mallei* irrespective of patient improvement [97].

All these findings agree with the described effect of an intracellular lifestyle resulting in an altered bacterial transcriptome, which may lead to a different morphology, a modified duplication time or an altered surface [107,108]. Of note, different studies have shown that the altered expression levels of genes apparently unrelated to the development of quinolone resistance may result in increased MIC levels to quinolones with a few genes increasing levels of quinolone efflux or impairing quinolone intake (see Section 8: Unconsidered Chromosomal Mutations).

Furthermore, the expression of specific genes seems to be relevant for the ability of bacteria to survive and multiply within amoebae [109]. Among these genes, it has been observed that the lack of functionality of *cmeB* impairs the survival and replication of amoeba-internalized *Campylobacter jejuni* [109]. CmeABC is an RND efflux pump present in the *Campylobacter* spp. genome, which is able to efficiently extrude quinolones, among other antibacterial agents, with reports showing that *cmeABC* overexpression promotes the selection of fluoroquinolone-resistant *C. jejuni* isolates [110,111]. Therefore, amoeba-fed resistance as well as active efflux of fluoroquinolones are co-selected characteristics of *Campylobacter*.

## 4. Bacterial Benthonic Lifestyle

Since the dawn of microbiology, antimicrobial susceptibility has been established in metabolically active microorganisms with a planktonic lifestyle. When susceptibility/resistance to any antimicrobial agent is reported in a clinical or veterinarian setting, this involves these planktonic microorganisms. Nonetheless, other scenarios may be present, including benthonic communities, almost imperceptible grown or real quiescent states (see Section 6.1: *Sporulation and Quiescence*).

Bacteria may organize sessile communities imbibed within a matrix conformed by exopolysaccharides, eDNA, proteins and other compounds which vary among species; furthermore, intraspecies variations in matrix composition have been observed, suggesting clonal/strain specificity [112,113]. This matrix hinders the action of several antibacterial agents by blocking their access to bacteria. This finding has been directly associated with enhanced tolerability/resistance to a series of antibacterial agents including tobramycin and vancomycin, among others [114,115,116]. These communities are the so-called biofilms, which are of special concern with respect to medical device contamination and chronic or prosthesis infections. Thus, while the surface-fixed and non-motile nature of biofilms often underlies prothesis- or device-related infections [117,118,119], biofilms are not immutable structures, and different stages of biofilm maturity and different pathways of bacterial release have been described [116,120,121], with this release of bacteria mainly contributing to systemic dissemination of infections as well as infection chronicity. In recent years, there has been increasing attention to bacterial benthonic communities, with a continuous growth of basic studies focused on the characterization of these communities. However, only a scarce number of studies have been focused on the impact of bacterial biofilms on patient outcomes and their influence on antibiotic treatment failure, strongly suggesting a chronic forgotten and disconsideration of their real relevance in clinical settings [117].

Regarding quinolones, minimal biofilm inhibitory concentrations (MBICs) and minimal biofilm eradication concentrations (MBECs) may exceed planktonic MICs and minimal bactericidal concentrations (MBCs), respectively, by >256-fold [122,123]. The routes by which bacterial biofilms enhance resistance to quinolones (or other unrelated antibacterial agents) are diverse and multifactorial and are mainly socially and intimately interrelated [114,124]. Furthermore, in the case of quinolones, the levels of resistance conferred by these mechanisms are additive to those related to the presence of the abovementioned canonical chromosomal and transferable mechanisms of resistance.

### 4.1. Biofilm Access

Classically, as mentioned above, the main reason for the enhanced levels of resistance to members of this antibacterial agent family has been considered to be impaired access of quinolones to biofilm, related to the difficulty in passing through the imbibing matrix. Notwithstanding, this issue is controversial. Thus, while in several studies the extracellular matrix does not seem to significantly affect the access of quinolones, such as ciprofloxacin, which are able to penetrate the biofilm and achieve internal biofilm concentrations that are higher than the usual planktonic MICs [125], other studies have described that the penetration of ciprofloxacin within biofilm was hindered [126]. In this scenario, it has been suggested that these differences might be related to factors including bacterial species, growth conditions and biofilm thickness [114].

In this sense, the final levels of quinolone resistance/tolerance in bacterial biofilm communities are related to the specific composition of the imbibing matrix. Thus, the presence of the Psl exopolysaccharide in the matrix of *P. aeruginosa* biofilms has been related to enhanced ciprofloxacin resistance [127]. To the contrary, the presence of Pel, another exopolysaccharide, which may be present in the *P. aeruginosa* biofilm matrix, plays no role in ciprofloxacin resistance/tolerance [128]. The mechanisms by which Psl affects ciprofloxacin activity remain unclear. Despite the charge-neutral nature of Psl, the eradication of biofilms when cationic antibacterial agents (i.e., polymyxin B, and tobramycin) were combined with NaCl allowed Billings et al. to propose, as a partial explanation for this phenomenon, that the Psl matrix can sequestrate these positively charged antibacterial agents by electrostatic interactions. However, this explanation was not extended to ciprofloxacin (negatively charged) as biofilms were not disaggregate when ciprofloxacin was combined with NaCl [127].

### 4.2. Altered Bacterial Metabolic Activity

The metabolic activity of bacteria living in benthonic communities is different from that of bacteria in planktonic status. Thus, reduced metabolic activity and differences in gene expression have been described [129,130].

A reduction in metabolic activity has been described as being a quinolone resistance/tolerance mechanism [131]. These antimicrobial agents need DNA Gyrase and Topoisomerase IV, which are responsible for DNA relaxing and duplication, to be active to exert their action (see Section 6.1: *Sporulation and Quiescence*).

The decrease in metabolic activity of bacteria imbibed within biofilms is related to different factors including oxygen concentrations and nutrient availability, which are greater or lesser according to whether they are in located in external biofilm surfaces or the most internal strata [132]. In biofilms, the most extreme reduction in metabolic activity is that related to bacterial quiescent status, with most dormant cells being deep within the biofilm (see Section 6.1: *Sporulation and Quiescence*). Of note, lower oxygen concentrations have also been related to lower levels of formation of reactive oxygen species (ROS), hypothesizing that lower ROS levels affect the bactericidal activity of quinolones [133].

In these scenarios, different bacterial systems are activated, including the so-called stringent response, or toxin/antitoxin modules (T/A) (see Section 6: Stringent Response and Toxin/Antitoxin Systems) [134].

In addition, differences in the transcriptomes of bacteria living in planktonic and benthonic communities have been described [129]. This finding results in differences in bacterial functionalities, which might also lead to alterations in bacterial resistance to antibiotics. In fact, different studies on quinolone-resistant mutants have reported the selection of apparently unrelated mutations as well as modifications in the transcriptome leading to increases in the final MICs to quinolones, despite the absence of established mechanisms of quinolone resistance (see Section 8: Unconsidered Chromosomal Mutations).

## 5. Nutrient-Independent Slow Growth

In 1996, it was shown that the presence of the plasmid pKM101 (belonging to the IncN incompatibility group) used in the de Ames test confers both a slow growth pattern and, in the presence of ciprofloxacin, a parallel increase in the survival of *Escherichia coli* growing on minimal media [135]. This slow-growth phenotype was related to a region of 2.2 kb including the *korB*, *traL*, *korA* and *traM* genes. The authors further hypothesized the role of the region surrounded by the *korB* and *korA* genes. The increased survival may be related to the mode of action of quinolones, which require the presence of active biological processes involving Topoisomerase activity [136,137]. Nonetheless, no data of the exact reasons for the slow growth associated with this plasmid have been provided, and to our knowledge, no further studies have been carried out.

## 6. Stringent Response and Toxin/Antitoxin Systems

Stringent response and the so-called toxin/antitoxin systems are bacterial systems involved in bacterial stress response [138,139].

Stringent response is a bacterial mechanism involved in a series of adaptations in response to different stress situations, such as nutrient or iron deprivation, oxidative stress, or others [140,141]. Meanwhile, toxin/antitoxin systems encode both a toxin able to interfere with different metabolic pathways leading to cell death or arresting cell growth and an antitoxin that inhibits the action of the toxin component [139]. To date, up to 8 T/A systems have been described, classified based on the mechanism of action of antitoxin component [141].

**Figure 2:**
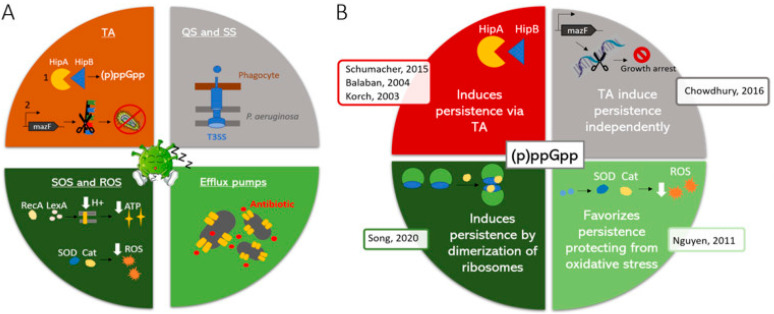
Proposed model of independent action of stringent and toxin/antitoxin systems. TA: Toxin/antitoxin system; QS: Quorum sensing; SS: Secretion systems; ROS. Reactive Oxygen Species; T3SS: Type 3 secretion systems. (**A**) Molecular mechanisms underlying bacterial persistence. (**B**) Different models explaining the involvement of (p)ppGpp in persistence and representative publications (see reference [135]). (**B**) red: T/A systems are activated by (p)ppGpp. grey: T/A systems induce quiescence independently; light green: (p)ppGpp protects against oxidative stress, favoring the development of persister cells; dark green: (p)ppGpp induces dimerization of ribosomes, which subsequently lead to persistence. References presents in the figure may be found at [138]. Reproduced from reference [138], with the permission of Elsevier.

Different proposals about how these metabolic routes drive towards dormant and quiescent bacteria have been made, including interdependent and independent routes of stringent response and T/A systems (Figure 2). Thus, while several authors propose that stringent response and T/A systems are two independent routes, it has also been proposed that the action of stringent response results in increased production of the Lon protein which, in turn, proceeds with the degradation of the antitoxin, allowing the toxin to exert its action, or the inverse scenario in which the inactivation of the antitoxin starts a series of metabolic processes leading to the overproduction of RelA, resulting in the activation of stringent response [138,142].

Stringent response is related to the action of a series of genes, including the *relA*, *spoT* and *dksA* genes [140,141]. The induction of stringent response results in the synthesis of the alarmones ppGpp or pppGpp (collectively referred to as (p)ppGpp) [143]. The impact of this mechanism on survival has been highlighted in studies in which mutants with impaired (p)ppGpp synthesis showed decreased survival in the presence of quinolones and lower MBC/MIC compared with parental isolates, while those with impaired hydrolysis of (p)ppGpp showed an opposite scenario [144].

It has been confirmed that a series of toxins from T/A systems, such as ParE toxin (not to be confounded with ParE, a subunit of Topoisomerase IV) or TisB from ParDE or TisAB-IstR-1, are released during ciprofloxacin exposure, favoring bacterial survival [145,146]. In the case of TisB, this finding is directly related to the presence of a Lex-box in its promoter region, which is activated by the SOS system that, in turn, is activated by DNA lesions related to the action of the quinolone [145]. On the other hand, increased levels of Lon have been claimed as the reason for the release of ParE [147]. Of note, there is currently controversy about the real role of T/A systems in the development of dormant cells [148].

### 6.1. Sporulation and Quiescence

**Figure 3:**
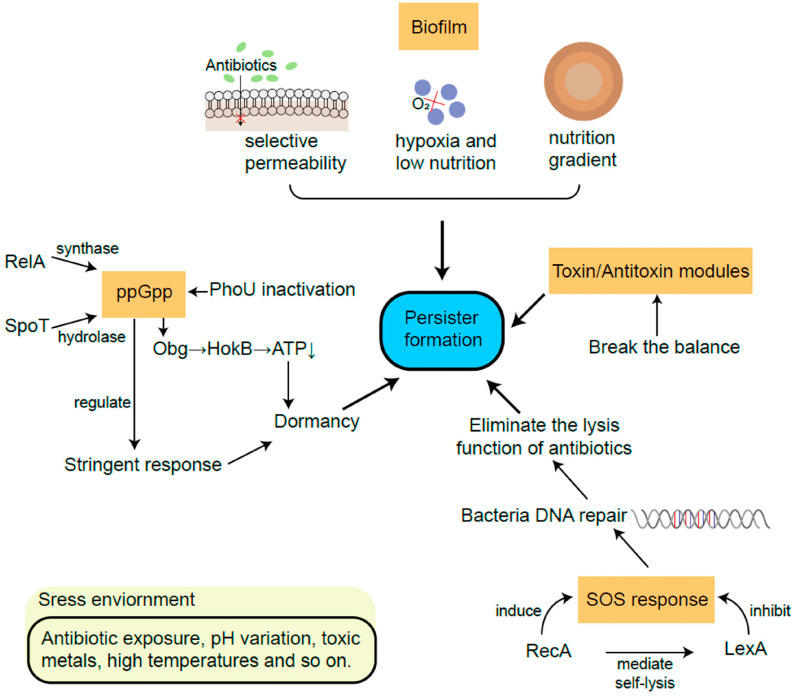
Models of development of persister cells. Note that while all pathways are represented as independent routes, different interactions among them may be present. Reproduced from reference [142], published under a Creative Commons license.

The abovementioned mechanisms led to the development of dormant bacteria (Figure 3). Sporulation and quiescence are conceptually related phenomena leading to long-term inactive or almost inactive microorganisms. Two notes: bacteria may remain viable for years in spore or quiescent states [149], and it should be considered that antibiotic susceptibility is restored when they are reactivated.

Sporulation is a bacterial mechanism of bacterial survival in extreme conditions in which the bacteria suffer a series of morphological alterations and enter into a truly dormant state, in which no metabolic process takes place until environmental conditions are more favorable [149]. Meanwhile, quiescence is another bacterial response to environmental stress. Quiescent microorganisms are in a viable non-replicating state, but in contrast to spores, quiescent bacteria display a basal metabolic process with no morphological alterations [149]. As mentioned previously, the quiescent state is also present within biofilm communities in bacteria living in the most internal biofilm layers [114].

DNA Gyrase and Topoisomerase IV, both classified as Type II Topoisomerases, are actively involved in different DNA processes, with the introduction of negative supercoiling and DNA decatenation of replication products as the most relevant and well established [55,150]. The quinolones need Topoisomerase activity to interact with its targets in order to block the DNA replication forks, generating cleaved DNA-Type II Topoisomerase complexes, and subsequently inducing rapid bacterial death through different protein synthesis-dependent or -independent pathways, with the SOS system possibly being involved in slow bacterial death [136,137].

In agreement, a series of studies have reported negative relations between bacteriostatic ribosomal-targeting antibacterial agents and different quinolones, ranging from indifference tending to antagonism, to declared antagonism leading to suppressive effects [131,136,151]. A related and paradoxical effect has been described in which bacterial survival at high nalidixic acid concentrations was higher than when the concentrations did not reach these values. This was explained by the bactericidal action of nalidixic acid being modified to bacteriostatic action, inducing a metabolic block of bacterial activity leading to a bacterial quiescent state [131]. Crumplin et al. [131] also showed that after exposure to >200 mg/L of nalidixic acid, the surviving *E. coli* K16 did not show higher nalidixic acid MIC than those at baseline. In accordance with this scenario, full bacterial dormancy in the sporulate and the almost inactive quiescent states allow bacteria to remain unaffected by the presence of quinolones, displaying higher resistance levels and increasing bacterial survival in the presence of these antimicrobial agents and, in summary, escaping the action of quinolones irrespective of their MIC to quinolones when reactivated.

## 7. Quinolone Inactivation/Modification

While not included in the present study because both AAC(6′)Ib-cr and CrpP (despite the aforementioned controversy) qualify as canonical mechanisms of quinolone resistance, the presence of numerous variants, and thus possible different spectra, affecting different quinolones or level of activity should be highlighted [1,76].

In addition to the largely described ability of AAC(6′)Ib-cr to inactivate several quinolones through acetylation [73,152], and the recently described CrpP proposed to be able to phosphorylate several quinolones, such as ciprofloxacin [153], a series of poorly described bacterial mechanisms of inactivation of quinolones are present in different microorganisms [1].

### 7.1. Fungi

Thus, in the 1990s, the ability of several wood-rotting fungi, including *Irpex lacteus*, *Gloeophyllum striatum* or *Phanerochaete chrysosporium,* among others, to degrade enrofloxacin was first described [154]. Thereafter, the ability of other fungi, such as *Clitocybe odora*, *Coriolopsis gallica, Cyathus stercoreus, Irpex lacteus*, *Xylaria longipes* or others, to degrade or modify quinolones has been largely described [155,156,157,158]. Of note, the resulting metabolites may retain a degree of antibacterial activity, which varies between processed quinolones and fungi species [156].

**Figure 4:**
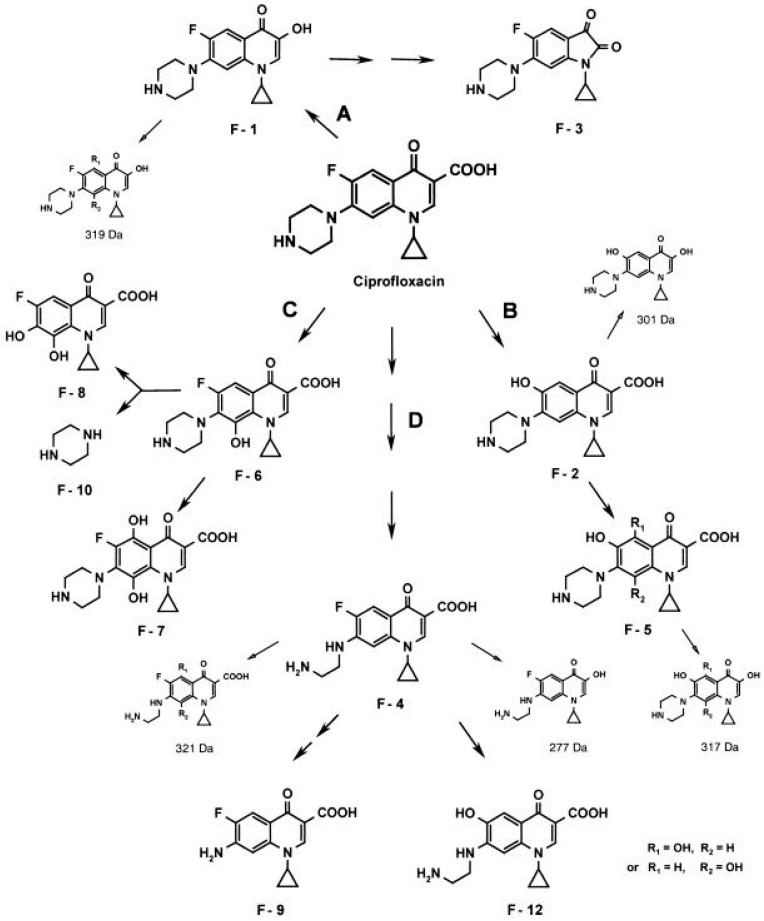
Proposed network of metabolites generated from ciprofloxacin by the brown rot fungus *Gloeophyllum striatum*. (**A**) Oxidative decarboxylation. (**B**) Defluorination. (**C**) Hydroxylation at C-8. (**D**) Oxidation of the amino moiety. Trace metabolites, detected only by HPLC–MS are represented at a reduced size. Reproduced from reference [158], with the permission of Elsevier.

The fungi degradation/modification routes of quinolones are diverse. Thus, working with *Gloeophyllum striatum*, Wetzstein et al. proposed four possible degradation routes: oxidative decarboxylation, defluorination, hydroxylation at C-8 or oxidation of the amino moiety (Figure 4) [158]. Data regarding the exact enzymes involved in these degradation/modification routes are scarce. Nevertheless, the role of enzymes such as cytochrome P450, or those possessing laccase-like and peroxidase-like activity, has been explored [155,156,159]; of note, the two latter types of enzymes are considered ligninolytic enzymes [160], in agreement with the apparent facility of wood-rooting fungi to degrade or modify quinolone. Along this line, a recent study analyzing the ability of *Coriolopsis gallica* to degrade levofloxacin suggested that enzymes with laccase-like and peroxidase-like activity may play a role due to their prevalence in fungi secretome [155]. Similarly, the chloroperoxidase of *Leptoxyphium fumago* (formerly *Caldariomyces fumago*) is also able to degrade norfloxacin [161]. On the other side, other authors have proposed that several peroxidases, such as for instance manganese peroxidases, play no role in the detoxification of quinolones, limiting this role to cytochrome P450 and laccases [159,162]. Regarding this latter issue, as mentioned previously, it should be considered that different amino acid sequences may result in different activity.

### 7.2. Bacteria

The ability of different bacteria to degrade or inactivate quinolones by non-canonical mechanisms of resistance has also been observed and may undoubtedly be considered as one of the factors underlying species-specific intrinsic levels of resistance to quinolones. Among the microorganisms with a demonstrated ability to degrade or inactivate quinolones, environmental bacteria, such as *Labrys portucalensis*, members of the genera *Mycobacteria* or *Microbacterium*, or extremofiles like members of the genus *Thermus,* among others, may be found [163,164,165,166]. Furthermore, the ability of relevant human pathogens to degrade or inactivate quinolones not related to canonical mechanisms of quinolones modification (i.e., AAC(6′)Ib-cr, CrpP) has also been reported, with descriptions of danofloxacin modifications by *P. aeruginosa* [167].

**Figure 5:**
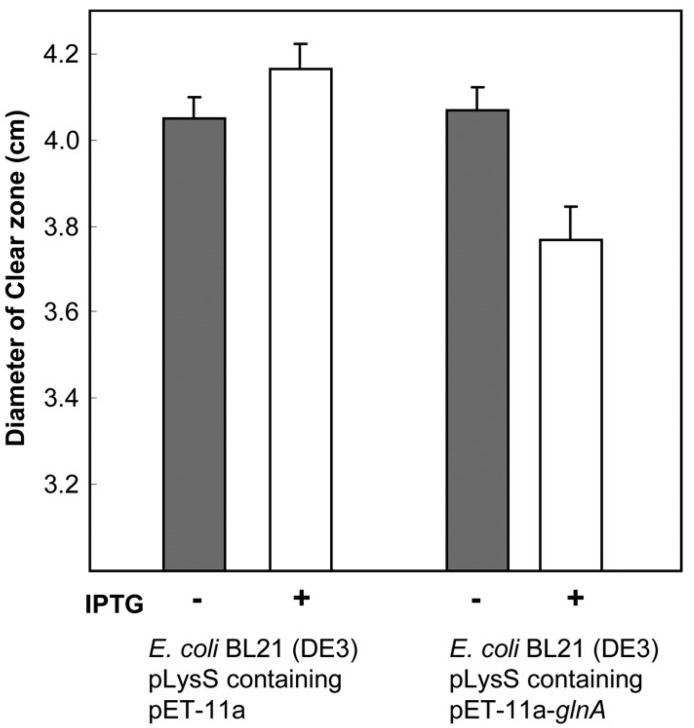
Effect of *glnA* on the susceptibility levels to norfloxacin. Norfloxacin disk assay of non-induced (gray bars) and IPTG-induced (white bars) cultures of *E. coli* BL21(DE3) pLysS containing plasmids with or without the *glnA* insert. In the cells with the *glnA* insert, the reduced diameter of the clear zones of inhibition due to induction of glutamine synthetase was statistically significant (*p* < 0.05). Reproduced from reference [168] with the permission of Elsevier.

As with fungi, there are a variety of quinolone-modifying routes. For instance, Kim et al. described four different norfloxacin modification routes in *Microbacterium*: hydroxylation, oxidative defluorination, desethylation and N-acetylation [165]. In 2013, the same group described that norfloxacin N-acetylation in *Microbacterium* was related to the action of a glutamine synthetase encoded in the gene *glnA* (GenBank access: JX901058), confirming this finding by cloning and expressing this gene in *E. coli*, and observing both the ability of the enzyme cloned in *E. coli* to modify norfloxacin and its effect on ciprofloxacin susceptibility [168] (Figure 5).

Nevertheless, the effect of glutamine synthetase on the final MIC of quinolones may also be influenced by a parallel effect on the production of OmpF related to the expression of *glnA*. Thus, an inverse relationship has been observed between the levels of expression of the *glnA* and *ompF* genes in *Salmonella enterica*, with lower levels of OmpF negatively affecting the intake of several quinolones, and thereby contributing to increasing the MIC levels to these agents [169].

SilA is another bacterial enzyme which has demonstrated its in vitro activity against different quinolones. Thus, Blanquez et al. cloned *E. coli* and purified SilA from the plant–pathogen *Streptomyces ipomoeae,* showing its ability to degrade ciprofloxacin and norfloxacin [170]. Of note, this enzyme also belongs to the laccases family [170]. Further in silico studies of the SilA protein have proposed that the amino acids His_102_, Val_103_ and Tyr_108_ are key in the modification of quinolones, with the first two being proposed to be involved in oxidative decarboxylation of the COOH radical at position 3, while Tyr_108_ is involved in the remotion of an oxygen for the carbonyl radical raised at position 4 [171]. A search in GenBank showed that SilA is present in a long series of microorganisms, with most having a high degree of identity with that of *S. ipomoeae*, including the critical amino acids proposed for anti-quinolone activity.

While almost all the abovementioned studies were focused on the biodegradation of quinolones in environmental or solid/liquid waste residues, it should be taken into account that all these quinolone-modifying mechanisms are at risk of mobilization to plasmids or other transferable genetic elements, as has been reported, for instance, with *qepA*, with the original source being proposed to be among *Comamonadaceae* [75], *qnrB,* which is indigenous of *Citrobacter freundii* [172], or *oqxAB*, chromosomally encoded in *Klebsiella pneumoniae* [173]. If this mobilization results in the lack of regulation, it will lead to a constitutive expression of the encoded protein and subsequently confer higher levels of resistance than in the original microorganism. As a general rule, all mechanisms of quinolone resistance are additive [1,55], and therefore, in a scenario in which *silA*, or any other gene encoding a protein with the potential of fully or partially inactivating quinolones, is mobilized to a plasmid—for instance by the action of insertion sequences—its exogenous acquisition by another microorganism should contribute to increasing its MIC levels to quinolones.

The fact that almost imperceptible amino acid alterations may expand or constrict the substrate profiles of enzymes able to introduce structural modifications, such as acetylation, phosphorylation or adenylation in antibacterial agents, as shown with AAC(6′)Ib [73], and the presence of a series of other fungi and bacterial chromosomal encoded proteins able, to a greater or lesser extent, to modify and degrade quinolones, suggest that new transferable quinolone-inactivating enzymes will be described in the forthcoming years.

## 8. Unconsidered Chromosomal Mutations

The selection of *gyrA* and/or *parC* mutations under quinolone exposure is a well-established phenomenon [44,50,80,81,174,175], with these mutations altering quinolone–DNA–DNA-Gyrase interactions [46,52,54]. Similarly, increased efflux levels as well as porin alterations resulting in decreased quinolone intake are also selected when microorganisms are exposed to quinolones, with both alterations resulting in lower cytoplasmic concentrations of quinolones and subsequent increased levels of antibiotic resistance [44,82,176]. While mutations at quinolone targets or either in regulator or constitutive genes related to efflux pumps or outer membrane proteins are clearly involved in the development of quinolone resistance, alterations in other genes outside the classical scope may induce subtle alterations. While these alterations produce slight changes in quinolone susceptibility levels, they do not modify the quinolone-susceptible status of microorganisms and remain unnoticed by classical methodologies, being considered as background noise or inter-strain MIC variations. However, these alterations can impact the ability of bacteria to develop full quinolone resistance. Of note, in multiple cases, these alterations induce modifications in different bacterial pathway regulation, which, over time, result in reduced quinolone uptake within the bacterial cytoplasm (Table 2).

In this regard, when analyzing the exposure to ciprofloxacin of *E. coli* J53 carrying cloned *qnr* (designed below as J53-*qnr*) determinants (*qnrA1*, *qnrA3*, *qnrB1* and *qnrS1*), Cesaro et al. showed that while no differences in the selection of mutants were observed, the mutant prevention concentration was ~10-fold higher than the original *E. coli* J53 [182]. Thus, the presence of *qnr* determinants facilitates the acquisition of quinolone resistance in the presence of higher antibiotic concentrations [182]. Surprisingly, while only 20% (65/329) of resistant isolates derived from J53-*qnr* presented mutation(s) in quinolone targets, this finding was observed in 79% (94/119) of quinolone-resistant mutants derived from *E. coli* J53, *p* < 0.0001 [182]. The authors suggested that Qnr blocks the access of quinolones to their targets, thereby favoring the selection of other mutations [182]. Other authors have observed similar phenomenon in *E. coli*, with Goto et al. also describing an increase in the mutant prevention concentrations as well as highlighting that parental strains acquire mutations in quinolone targets earlier than their derived strains with cloned *qnr* genes [183]. Vinué et al. expanded the scenario to other TMQRs, such as *qepA* and *aac(6′)Ib-cr* [177].

### 8.1. Toxic Metabolites Accumulation

These apparently unrelated mutations which are selected in this scenario were explored in different studies, with some probably being directly or indirectly involved in the regulation of efflux pump or porin expression levels, thereby resulting in the action of canonical mechanisms of resistance. For instance, alterations impairing *cysH* (cysteine biosynthesis), *icdA* (tricarboxylic acid cycle), *metE* (methionine synthesis) or *purB* (adenine biosynthesis) gene expression have been involved in metabolite accumulation-induced *acrAB-tolC* activation, increasing efflux and resistance levels to the tested quinolone, nalidixic acid [178]. While the metabolites related to these genes are different and belong to different metabolic processes, they lead to a common scenario, strongly suggesting the activation of efflux pumps to pump out toxic accumulations of intermediate products [178]. Of note, a possible association between *purB* mutations and quinolone resistance was suggested as early as 1970 [184]. On the other side, Vinué et al. also selected an in vitro ciprofloxacin-resistant *E. coli* mutant with an impaired *icdA* because of an *IS*10 insertion, but it was not correlated with increased levels of AcrAB-TolC [177].

### 8.2. RNA Polymerase

RNA polymerase subunits have also been involved in the development of low levels of quinolone resistance, with the *rpoA* mutation N_294_Y being observed in 4 *S. enterica* mutants [179]. While selected concomitantly with mutations in other genes, including well-established *acrAB-TolC* regulator genes, the cloning of the mutant *rpoA* results in final increases in the MICs of nalidixic acid (from 2 mg/L to 32 mg/L) and ciprofloxacin (from <0.015 mg/L to 0.015 mg/L) [179]. Regarding another subunit, the easy selection of *rpoB* mutations after ciprofloxacin exposure has been highlighted, with 20–30% of selected mutants carrying an *rpoB* alteration [180]. Thus, Pietsch et al. observed the presence of the amino acid substitutions H_1244_L, E_1272_G, A_1277_V, E_1279_G, Δ_442–445_ and duplication of the S_455_ (DuS_445)_ in *E. coli* exposed to ciprofloxacin in vitro [180]. The authors cloned and analyzed three of the *rpoB* mutations (those leading to the changes in the amino acids A_1277_V, Δ_442–445_ and DuS_445_), observing increases from 0.015 mg/L to 0.023–0.045 mg/L in the MIC to ciprofloxacin [180]. Furthermore, the additive effect on the final MIC to ciprofloxacin was also observed when alterations in established quinolone resistance determinants (e.g., *gyrA*, *gyrB, soxR*, *marR* or *acrR*) were also present [180]. In addition, a very modest effect (maximum MIC of 48 mg/L) of these three amino acid changes in the MIC of rifampicin was also generated [180]. When the impact on the expression of other genes of the six *rpoB* mutations was determined, the authors observed that more than 100 genes were affected, including increased levels of *mdtK* expression, an efflux pump encoding gene, in all six cases, with further analysis suggesting a direct relationship between the presence of *rpoB* mutations and overexpression of *mdtK* [180]. Meanwhile, using *qnrA1*-carrying *E. coli* J53, Vinué et al. described that the *rpoB* mutation R_1246_C increased the MIC levels of nalidixic and ciprofloxacin from 8 mg/L to 64 mg/L and from 0.016 mg/L to 1,5 mg/L, respectively [177]. The *rpoB* mutant showed a significant decreased expression of *ompF*, thereby impacting quinolone intake within the bacterial cytoplasm [177]. Of note, while no effect on fitness was observed by Vinué et al., the opposite scenario was reported by Pietsch et al., a finding that might be related to the final alteration induced [177,180]. While not further explored, additional mutations in *rpoB* and *rpoC* have also been reported in ciprofloxacin-resistant *E. coli* mutants as well as in ciprofloxacin-resistant animal isolates [181,185].

### 8.3. Aminoacyl-tRNA Synthetase Genes

The presence of mutations in aminoacyl-tRNA synthetase *aspS* (D_207_A), *leuS* (L_41_H, D_162_N, S_496_P) and *thrS* (H_244_P, I_582_S) genes has also been involved in the development of low levels of resistance in *E. coli* exposed to ciprofloxacin [181]. Centering the study on *leuS* D_162_N, S_496_P amino acid substitutions, MIC studies suggest that alone these mutations play no role in the final MIC levels when introduced into strains carrying concomitant mutations in well-recognized quinolone targets, leading to a slight additive effect on final resistance levels [181]. The authors highlighted that the presence of these mutations influenced the final expression levels of around 200 genes and proposed the induction of a *relA*-related stringent response, in at least *aspS* and *leuS* mutants. This response leads to the overexpression of different genes, such as *mdtK*, encoding an efflux pump, *acrZ*, related to the efflux pump AcrAB-TolC or *ydhIJK*, a putative efflux pump [181]. Nevertheless, the scenario is highly complex with apparently contradictory data including the overexpression of *acrA* but the underexpression of *acrB*, or the contradictory overexpression of *ompF* (which theoretically leads to increased quinolone intake) [181]. The presence of a massive number of proteins expressed differently between quinolone-resistant and quinolone-susceptible microorganisms has also been described in other studies [186].

### 8.4. Mutations in Other Targets

While to the best of my knowledge no study on quinolones has been carried out, the deletion from amino acid 82 to 84 in the ribosomal protein L22 has been proposed to be related to an AcrAB-TolC overexpression [187]. Although further analysis is needed, if the impact of this deletion on AcrAB-TolC levels were definitively established, it would also result in modifications in the final cytoplasmatic quinolone levels and would thereby impact the final MIC to quinolones.

In addition, mutations in a series of other genes have been found when mutants were selected in the presence of quinolones, but the possible effect of these mutations on the final MIC levels remains unclear [177]. In this sense, the mutagenic power of quinolones must be considered [188,189].

Further studies on gene inactivation by the insertion of a transposon have shown that the inactivation of tens of genes (other than those classically considered) slightly increases or decreases ciprofloxacin susceptibility levels by 2-fold (in general) [190], and this might, therefore, underlie subtle differences in the basal levels of quinolone resistance. This finding demonstrates that point mutations in several of these genes cannot be discarded as actually additional contributors to the enhancement of quinolone resistance levels.

Vinué et al. suggested that the presence of the low levels of resistance conferred by a TMQR favors the development of a series of mutations, which, despite only having a low impact on the final susceptibility, altogether result in the development of full quinolone resistance [177]. While apparently unrelated, it has been shown that *P. aeruginosa* isolates carrying the *exoU* gene have an enhanced ability of acquiring high levels of resistance to quinolones and multiple mutations in *gyrA* and *parC*, while in a percentage of *exoU*- isolates, the acquisition of resistance to quinolones seems to be related to other pathways, including efflux pump overexpression, and the presence of isolates possessing high levels of resistance is significantly lower than among ExoU+ isolates [191,192,193]. This finding has been explained by the lower fitness cost of *P. aeruginosa exoU*+ related to the development of quinolone target mutations [191,192,193]. This fitness cost may better explain the abovementioned phenomenon observed by [177] Vinué et al. Thus, it can be hypothesized that these apparently unrelated mutations have no fitness cost, or the imposed fitness cost is lower than that related to quinolone target mutations. Isolates presenting a TMQR only need a slight increase in the MIC levels to become fully resistant to quinolones (i.e., survive and duplicate in the presence of quinolone concentrations achieved in their surrounding environment). Meanwhile, isolates which do not have this initial advantage need to become resistant with the presence of target mutations, irrespective of the fitness cost, and mutations in “non-canonical” genes may appear in a second phase.

Further analysis designed to obtain greater knowledge of the interactions of this constellation of punctual mutations and up- and downregulate genes and their derived effects is needed to better understand this phenomenon and clarify which genes play a role in the development of quinolone resistance and which do not.

### 8.5. Small Colony Variants

While different studies have not shown increased levels of quinolone resistance and, in fact, sometimes show slight increases in quinolone susceptibility [194,195], the SCVs have also been related to increased levels of quinolone resistance and enhanced persistence, even in the absence of target mutation [90,196,197]. SCVs are naturally occurring variants described in both Gram-negative and Gram-positive bacteria, such as *Burkholderia pseudomallei*, *Coxiella burnetii*, *E. coli*, *P. aeruginosa* or *S. aureus*, with an apparently unvaried morphology but exhibiting both a small size and a higher duplication time than their “normal” counterparts [90,194,196,198,199]. It has been proposed that these characteristics are related to the presence of a series of chromosomal mutations, leading to an altered electron-transport chain often resulting in menadione and/or hemin auxotrophy, or to the presence of thymidine auxotrophy [195,200,201,202,203], as well as other mutations, such as in the Pf1 prophage region [194]. Furthermore, the presence of genetic rearrangements as SCV inductors has also been described [204]. These alterations result in additional transcriptomic modifications [203], which might differ between different mutants presenting an SCV phenotype and thereby underlie the abovementioned differences related to the effect of quinolones on SCVs [205]. In this sense, alterations in the outer membrane and altered production of GyrA have been proposed as plausible explanations [90,197].

### 8.6. Atypical Amino Acid Substitutions in GyrA

Finally, while the classical quinolone resistance hot spot of the amino acid codon Ser_83_ of GyrA is affected [54], the rareness of the Ser_83_STOP (amber codon) mutation requires a brief comment [177,182]. Although this mutation should be lethal because of the essential role of the DNA-Gyrase, several microorganisms dispose of a “rescue system” for premature stops, the so-called tRNA suppressors, which recognize the STOP codon and insert an amino acid [206]. Regarding both of the abovementioned studies, Vinué et al. described the presence of an amber suppressor, while Cesaro et al. hypothesized the same option [177,182]. The presence of an amber suppressor may lead to the introduction of tryptophan, leucine or tyrosine, among other amino acids [206], with all three mentioned amino acids leading to quinolone resistance when raised in position 83 of GyrA [34,42,55,207]. Of note, the efficiency of these suppressors is lower than normal [206], and therefore, the presence of a lower amount of GyrA is predictable, which, as commented above, might also impact, by itself, the final quinolone resistance levels. Of note, a similar rescue system operates for ochre (TAA) and opal (TGA) terminators [206].

## 9. Unknown Mechanisms

The presence of undescribed mechanisms of quinolone resistance, either classified within canonical mechanisms, for instance as new transferable enzymes able to modify and inactivate one or more quinolones, or not, as in mutations in other apparently unrelated genes, cannot be ruled out, independently of the level of resistance conferred, or the extension and clinical relevance. In this sense, Chavez-Jacobo et al. noted that the original plasmid containing the *crpP* gene was able to confer higher levels of ciprofloxacin resistance than in vitro recombinant plasmids carrying *crpP*, thereby hypothesizing the presence of an additional undescribed plasmid-encoded mechanism of quinolone resistance [153].

## 10. Conclusions

While canonical mechanisms of quinolone resistance are undoubtedly the mechanisms most frequently described in quinolone-resistant microorganisms [55], a series of mechanisms and lifestyles may result in a low level of resistance to quinolones or in a quinolone escape route for microorganisms. Several of these mechanisms, as chromosomal mutations out of classical targets, may underlie the first steps towards the development of quinolone resistance and may thereby be selected in the presence of low concentrations of these agents. In this sense, the fitness cost of some of these mutations seems to be lower than that related to quinolone target mutations. This finding is of relevance in the microbial world beyond hospital settings, as natural environments in which quinolone residues arrive due to anthropogenic actions favor the initial selection of microbial populations with decreased susceptibility to quinolones, which, in turn, may facilitate the selection of full quinolone-resistant microorganisms. Meanwhile, at the clinical level, the possibility of the mobilization of these mechanisms, or at least of those involved in quinolone inactivation, is the most relevant risk.

A better understanding of these mechanisms may contribute to the fight against quinolone resistance and, by extension, to the struggle against antimicrobial resistance.

## Figures and Tables

**Table 1:** Main unconsidered mechanisms of resistance/resilience to quinolones.

	Cause of Resistance/Tolerance					
	Low Levels of Targets	Decreased Antibiotic Access	Transcriptomic/Metabolic Alterations	Quinolones Inactivation/Degradation	Resistance	Tolerance
Expression levels of targets	Y	NA	NC	--	Y	--
Amoeba protection	NC	Y	Y	NA	Y	Y
Biofilm	NC	V	Y	--	--	Y
Nutrient-Independent Slow Growth	NC	NC	NC	--	--	Y
SR and T/A Systems ^1^	NC	NC	Y	NA	--	Y
Quinolones’ modification ^2^	--	--	--	Y	Y	--
Chromosomal mutations ^3^	Y	Y	Y	NA	Y	--

Y: Yes; NA: No data available; NC: Possible but not confirmed; V: Variable. ^1^ Stringent Response and Toxin/Antitoxin Systems. ^2^ Exclude AAC(3′)Ib-cr and CrpP. ^3^ Exclude mutations at *gyrA*, *gyrB*, *parC* and *parE* genes, excepting those leading to premature STOPs.

**Table 2:** Non-classical chromosomal mutations.

		Mutation						
Gene	Microorg.	D ^1^	S ^2^	Protein Encoded	Alt. ^3^	Effect ^4^	Impact ^5^	Reference
*icdA*	*E. coli*	*Tn*	*IS*10	Isocitrate dehydrogenase	LF	MA	↑ *acrAB-tolC* ^6^	[177,178]
*purB*	*E. coli*	*Tn*	--	adenylosuccinate lyase	LF	MA	↑ *acrAB-tolC*	[178]
*cysH*	*E. coli*	*Tn*	--	3′-phosphoadenosine 5′-phosphosulfate (PAPS) sulfotransferase	LF	MA	↑ *acrAB-tolC*	[178]
*metE*	*E. coli*	*Tn*	--	homocysteine methyltransferase	LF	MA	↑ *acrAB-tolC*	[178]
*rpoA*	*S. enterica*	--	N_294_Y	RNA polymerase subunit	TBC	TBC		[179]
*rpoB*	*E. coli*	--	R_146_C, H_1244_L, E_1272_G, A_1277_V, E_1279_G, Δ_442–445_, DuS_445_	RNA polymerase subunit	TBC	TBC	↓ OmpF, ↑ MdtK	[177,180]
*leuS*	*E. coli*	--	L_41_H, D_162_N, S_496_P	aminoacyl-tRNA synthetase (Leu)	RA	SR	↑ MdtK; ↑ *ydhIJK*	[181]
*aspS*	*E. coli*	--	D_207_A	aminoacyl-tRNA synthetase (Asp)	TBC	SR		[181]
*thrS*	*E. coli*	--	H_244_P, I_582_S	aminoacyl-tRNA synthetase (Thr)	TBC	TBC		[181]
*gyrA*	*E. coli*	--	Ser_83_STOP	DNA-Gyrase subunit	RE	TBC	↓ GyrA	

Microorg: Microorganisms; *Tn*: Transposon; LF: Loss of function; TBC: To be confirmed; RA: Reduced activity; RE: Reduced expression; MA: Metabolites accumulation; SR: Stringent response. ↓ Decresed levels of protein/expression of genes. ↑ Increased levels of protein/expression of genes. ^1^ D: Direct mutagenesis: Studies using Transposons (Tn) or similar to disrupt genes. ^2^ S. Selected mutants: Studies on quinolone-resistant microorganisms selected by growing on antibiotic- or biocides-containing plates. ^3^ When known, resulting alteration. ^4^ When known, effect of described alteration. ^5^ Proposed impact on established quinolone resistance mechanisms. Other impacts in known and/or unknown quinolone resistance mechanisms may not be discarded. ^6^ An alternative pathway might be present following the Vinué et al. results [177].

## Data Availability

No new data were created or analyzed in this study. Data sharing is not applicable to this article.
